# A Web-Based Platform (CareVirtue) to Support Caregivers of People Living With Alzheimer Disease and Related Dementias: Mixed Methods Feasibility Study

**DOI:** 10.2196/36975

**Published:** 2022-08-04

**Authors:** Justin J Boutilier, Priya Loganathar, Anna Linden, Eleanore Scheer, Sofia Noejovich, Christian Elliott, Matthew Zuraw, Nicole E Werner

**Affiliations:** 1 Department of Industrial and Systems Engineering University of Wisconsin-Madison Madison, WI United States; 2 Department of Engineering Systems and Environment University of Virginia Charlottesville, VA United States; 3 Whiplash Technology, Inc Palm Springs, CA United States; 4 Department of Health and Wellness Design Indiana University School of Public Health-Bloomington Bloomington, IN United States

**Keywords:** Alzheimer disease and related dementias, mHealth, caregivers, dementia caregiving, eHealth, telehealth

## Abstract

**Background:**

People living with Alzheimer disease and related dementias (ADRD) require prolonged and complex care that is primarily managed by informal caregivers who face significant unmet needs regarding support for communicating and coordinating across their informal care network. To address this unmet need, we developed CareVirtue, which provides (1) the ability to invite care network members; (2) a care guide detailing the care plan; (3) a journal where care network members can document, communicate, and coordinate; (4) a shared calendar; and (5) vetted geolocated caregiver resources.

**Objective:**

This study aims to evaluate CareVirtue’s feasibility based on: (1) Who used CareVirtue? (2) How did caregivers use CareVirtue? (3) How did caregivers perceive the acceptability of CareVirtue? (4) What factors were associated with CareVirtue use?

**Methods:**

We conducted a feasibility study with 51 care networks over a period of 8 weeks and used a mixed methods approach that included both quantitative CareVirtue usage data and semistructured interviews.

**Results:**

Care networks ranged from 1 to 8 members. Primary caregivers were predominantly female (38/51, 75%), White (44/51, 86%), married (37/51, 73%), college educated (36/51, 71%), and were, on average, 60.3 (SD 9.8) years of age, with 18% (9/51) living in a rural area. CareVirtue usage varied along 2 axes (total usage and type of usage), with heterogeneity in how the most engaged care networks interacted with CareVirtue. Interviews identified a range of ways CareVirtue was useful, including practically, organizationally, and emotionally. On the Behavioral Intention Scale, 72% (26/36) of primary caregivers reported an average score of at least 3, indicating an above average intention to use. The average was 81.8 (SD 12.8) for the System Usability Scale score, indicating “good” usability, and 3.4 (SD 1.0) for perceived usefulness, suggesting above average usefulness. The average confidence score increased significantly over the study duration from 7.8 in week 2 to 8.9 in week 7 (*P*=.005; r=0.91, 95% CI 0.84-0.95). The following sociodemographic characteristics were associated with posting in the journal: retired (mean 59.5 posts for retired caregivers and mean 16.9 for nonretired caregivers), income (mean 13 posts for those reporting >US $100K and mean 55.4 for those reporting <US $100K), relationship to care recipient (mean 18.7 posts for child and mean 56.4 for partners/spouses), and living situation (mean 44.7 for those who live with the care recipient and mean 13.1 for those who do not). Older care recipients were associated with fewer posts (r=–0.33, 95% CI –0.55 to –0.06).

**Conclusions:**

This study establishes the acceptability and feasibility of CareVirtue among ADRD care networks and highlights the importance of designing flexible, multicomponent interventions that allow care networks to tailor their engagement according to their needs. The results will be used to improve CareVirtue feasibility and acceptability in preparation for a subsequent randomized trial to test CareVirtue’s effectiveness in improving caregiver outcomes.

## Introduction

### Background

More than 6 million individuals in the United States are living with Alzheimer disease and related dementias (ADRD) and it is attributed as the cause of death for 1 in 3 individuals over the age of 65 [1]. In the past 2 years, deaths attributed to ADRD have increased by 16% and research has projected that the number of people living in the United States with ADRD will triple by 2060 [1].

Individuals living with ADRD require prolonged and complex care that is primarily managed by informal caregivers. Informal caregivers are unpaid, nonprofessionals that provide care and typically include family and friends. There are an estimated 11 million caregivers providing care for people living with ADRD in the United States and they provide approximately 15.3 billion hours of unpaid care valued at nearly US $257 billion [2]. Caregivers report being undertrained, under-supported, and under-resourced to perform their caregiving role. Although caregivers can experience positive outcomes related to caregiving, the imbalance of caregiving demands and supports is often associated with mental, physical, and economic challenges that can lead to significant consequences for caregivers and the individual living with ADRD, such as caregiver stress, burden, depression, and morbidity [3-5].

To address these suboptimal caregiver outcomes, the US National Institute on Aging and other national advisory panels have highlighted the development and testing of technology-based interventions for caregivers of people living with ADRD as a key priority [6-8]. For example, the 2015 Alzheimer’s Research Summit highlighted the need to “test the use of technology to overcome the workforce limitations in the care of older adults with dementia as well as providing caregiver support and education.” [7]. In response, researchers have developed numerous information technology interventions such as mobile apps and websites to support ADRD caregivers across a range of domains including caregiver education, self-care support, support for managing behavioral symptoms of dementia, and virtual peer support groups [9-11]. Several systematic reviews and recent meta-analyses report that these technology interventions can improve outcomes for caregivers, such as increased self-efficacy and reduced ADRD caregiver burden, stress, and depression [3,12-19]. These reviews also suggest that effective interventions offer multiple components, tailored options, and social support [9-11].

However, a significant gap in existing interventions is that most focus only on the primary caregiver, even though most people living with ADRD receive care from more than 1 caregiver—*a care network*—with varying degrees of involvement [20-24]. Currently, caregivers face significant unmet needs regarding support for communicating and coordinating across the care network including sharing information, maintaining situation awareness, distributing responsibilities, scheduling, and managing caregiver hand offs [20-24].

Although some mobile apps exist that allow caregivers to share information, they are limited in their functionality, quality, and potential to meet the specific needs of ADRD caregivers [25]. A recent review of mobile apps for caregivers of people living with ADRD available on the US market identified 2 mobile apps that support shared communication and coordination [25]. According to study findings, one of those apps did not function consistently and received a quality rating of inadequate as indicated by the Mobile Application Rating Scale (MARS). The second app received an overall quality rating of minimally acceptable quality according to the MARS but scored lower than average on subjective quality. A similar study conducted in 2018 by Wozney et al [26] identified 3 mobile apps that connect a primary caregiver to other members of the care network. One of the apps identified is no longer available on the US market. The other 2 apps identified are not specifically designed to meet the needs of caregivers for people living with ADRD and are limited in their function (eg, only provide a shared calendar).

To address the current gaps in existing interventions, we developed CareVirtue, a progressive web application developed in React to support and connect ADRD care networks that can be accessed via a web browser on any device with a data connection. CareVirtue seeks to address the current gaps in existing ADRD caregiver support technologies through a high-quality, user-centered, ADRD caregiver–specific multicomponent technology to support communication, coordination, and connection among care networks. CareVirtue was initially inspired by an online support group for people newly diagnosed with ADRD and their caregivers in which support group members expressed an unmet need for tools to support communication and coordination among the care network. The need for CareVirtue was further supported by several findings from foundational research on care networks. First, findings from Block et al [27] suggested that primary caregivers require technologies to communicate and coordinate among the care network, that they try to adapt existing technologies (eg, email, messaging) to meet their needs, and that adaptation requires additional time and effort. Further, Ponnala et al [20] found that for primary caregivers, the currently under-supported communication and coordination among the care network increases their caregiving demands. Moreover, Tang et al [24] highlighted the consequences caregivers experience with under-supported care network communication and coordination, including maintaining situation awareness among caregivers, missing care information leading to potential patient harm (eg, missing a medication dose), and miscommunication leading to care network tensions or conflict.

Collectively, this prior research provides the foundation for CareVirtue. CareVirtue’s design honors the person-centered care model for people living with ADRD and their caregivers by (1) treating people living with ADRD as individuals with unique needs; (2) seeing the world from their perspective; and (3) creating a positive social environment in which the person living with ADRD and caregiver can experience relative well-being and quality of life. CareVirtue was designed and developed through consistent, iterative user input across multiple stages of usability testing coupled with expert evaluation. CareVirtue was specifically designed to encapsulate the foundational principles of person-centered care through the following features. Multimedia Appendix 1 provides a detailed walkthrough of CareVirtue features, which are given in brief in Textbox 1.

**CareVirtue features.**

**CareVirtue Dashboard**

**Figure 1:**
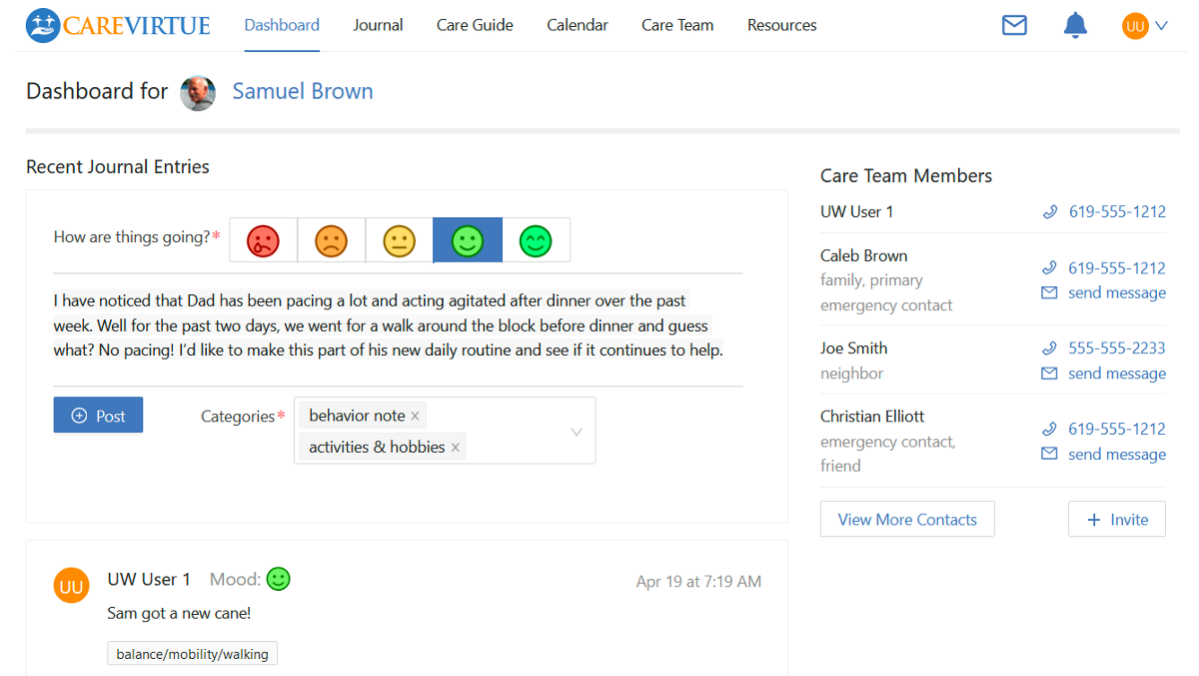
The CareVirtue Dashboard, a centralized hub to document and share important information with the care team.

Acts as a centralized hub to document and share important information with the care team. The dashboard includes a view presenting upcoming care appointments and events, linked to the care calendar; a list of current and pending care team members; and a journal where care network members can document, communicate, and coordinate about daily care events (Figure 1).

**Journal Reports**

Search and filter options to explore trends and gain insights into care recipient needs. The care journal or portions of the care journal can be exported to PDF to share as necessary.

**Care Guide Template**

**Figure 2:**
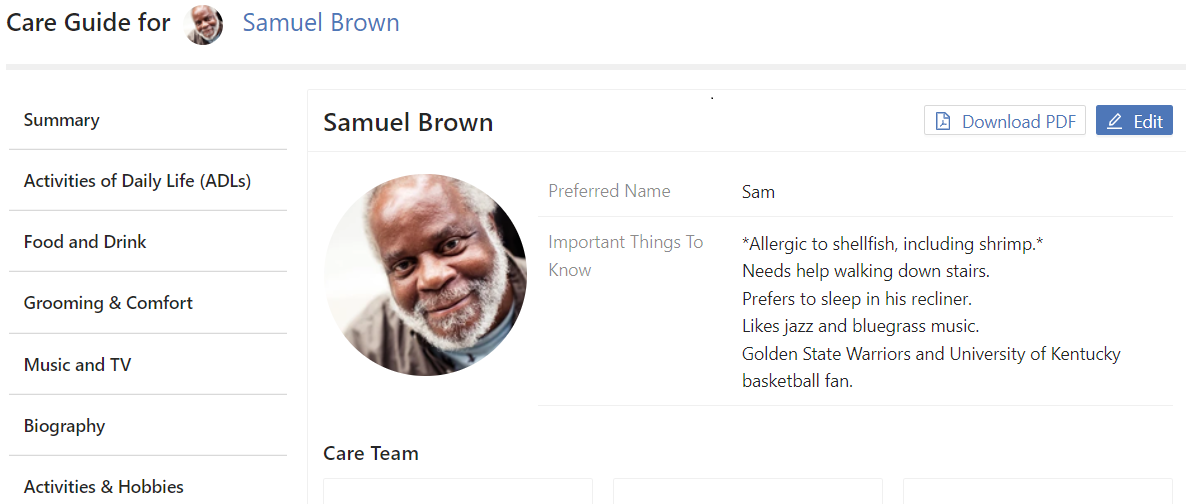
The CareVirtue Care Guide.

Includes a table of abilities related to specific activities of daily living and instrumental activities of daily living, and sections for needs and preferences, with a focus on quality-of-life details to help any caregiver understand the care recipient as a whole person (Figure 2). See Multimedia Appendix 2 for a detailed version of the care guide template.

**Care Team Management**

**Figure 3:**
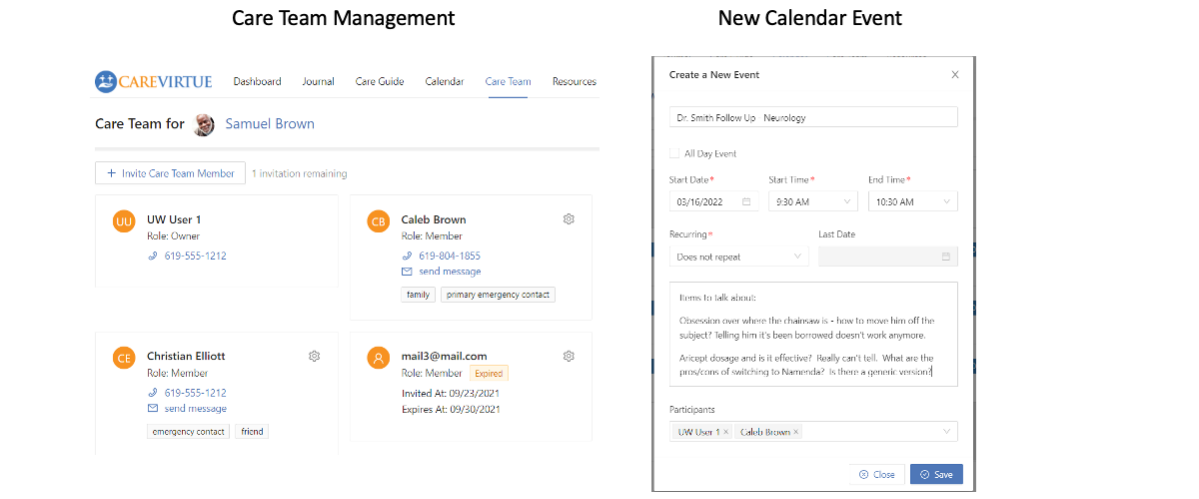
The CareVirtue Care Team management feature and form to create a new calendar event.

The ability to invite care network members to use the account with the primary caregiver with security permissions assigned at each invitation (Figure 3).

**Shared Calendar**

Supports scheduling and sharing recurring care events, reminders, and appointments (Figure 3).

**Geolocated Resources List**

**Figure 4:**
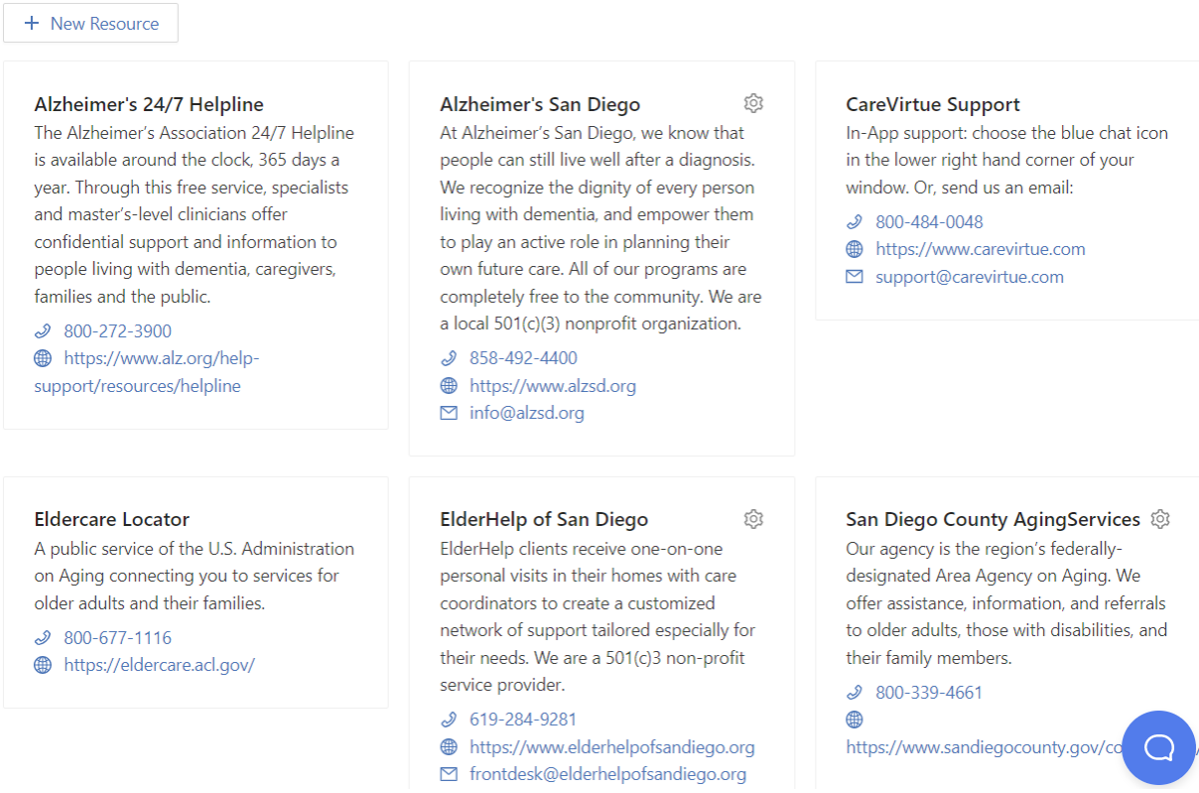
Example of the CareVirtue resources list for a caregiver living in San Diego, California.

For the current study, resources were limited to the Alzheimer’s Association 24×7 helpline, contact details for CareVirtue support, and contact details for the research team. The subsequent version of CareVirtue will include caregiver and person living with ADRD resources specific to their specific location such as the local area agency on aging (Figure 4).

### Objectives

In this study, we used a mixed methods approach to evaluate CareVirtue’s feasibility across the following research questions:

- Who used CareVirtue?
- How did caregivers use CareVirtue?
- How did caregivers perceive the acceptability of CareVirtue?
- What factors were associated with CareVirtue use?

## Methods

### Design

We conducted a feasibility study over a period of 8 weeks with the purpose of demonstrating CareVirtue’s acceptability and feasibility among care networks of people living with ADRD. This study reports one aim of a larger project, which has 2 specific aims. The first aim is the focus of this study. The second aim is to leverage the CareVirtue data generated by this feasibility study to develop an intelligent caregiver assistant (R41AG069607). The larger sample size and longer study duration than is typical for feasibility studies is due to adjustments made to achieve the second goal [28].

### Setting and Sample

Participants were recruited between February and June 2021 through multiple community sites in Wisconsin and Southern California as well as through the Wisconsin Alzheimer’s Disease Research Center. Advertisements for study participation were distributed via email, social media, and newsletter posts. Interested individuals contacted the study team via email or phone and were subsequently phone screened for the following eligibility criteria: self-identified primary caregiver of a person living with ADRD, at least 18 years of age, English speaking, daily internet access, and shares caregiving information/responsibility with other caregivers.

### Procedures

Eligible participants were scheduled for a 1-hour enrollment visit via videoconferencing software. During the enrollment visit, a study team member obtained informed consent from the primary caregiver and from the associated person living with ADRD (ie, the care recipient). If the person living with ADRD did not have decisional capacity to consent, the primary caregiver could consent on his/her behalf if he/she was the legally authorized representative. Next, the study team member administered a pretrial demographic survey, helped create the CareVirtue account, and provided a walk-through of CareVirtue’s functionality. Primary caregivers selected and invited secondary caregivers (to form a care network) at their own discretion. Once secondary caregivers were invited, they were separately contacted via email to electronically obtain informed consent.

Following enrollment, participants used CareVirtue for 8 weeks. During the use period, we administered a weekly survey starting 1 week after enrollment to assess caregiver workload and confidence using CareVirtue. If the survey was not completed, a follow-up reminder was sent the following day. At the completion of 8 weeks of use, we conducted a posttrial visit with the primary caregiver participant via Zoom where we administered posttrial surveys and conducted a semistructured interview as described in the following section. Primary caregivers were provided with a US $150 e-gift card at the end of the study period. Secondary caregivers contacted the study team if they were interested in participating in the postuse survey and interview. Secondary caregivers received US $15 for completion of the postuse survey and US $25 for completion of the 30-minute postuse interview.

### Data Sources

#### Demographic Data

We collected primary caregiver characteristics including age, gender, race and ethnicity, income, education, marital status, location, and employment. We also collected demographics for the care receiver including age, gender, ethnicity, living situation, and relationship to the primary caregiver (Table 1). Demographic data were not collected from the secondary caregivers.

#### CareVirtue Use

We collected data on 8 CareVirtue platform usage metrics across the study period: number of log-ins, journal posts, journal post replies, calendar events, secondary caregiver invites sent, secondary caregiver invites accepted, care guide sections created, and resources accessed. The log-in data are not fully representative of actual use because users could remain logged in to CareVirtue depending on their preference to log out.

#### Acceptability Measures

##### Quantitative Acceptability Measures

To assess acceptability, we measured primary caregiver perceptions of usability and usefulness. We measured usability using a weekly confidence survey with a single question: “rate your confidence in using CareVirtue on a scale from 1 (not at all confident) to 10 (very confident)” and using the System Usability Scale (SUS), which includes 10 statements (eg, “Learning to use CareVirtue was quick for me”) and a 5-point response scale (1=strongly disagree to 5=strongly agree) [29]. We measured usefulness with 3 surveys. First, we used the National Aeronautics and Space Administration-Task Load Index (NASA-TLX) to assess caregiver workload on a 6-item subscale with a 100-point range (1=very low to 5=very high). The purpose of this measure was to understand the relationship between caregiver workload and CareVirtue use. Second, we used the Behavioral Intention Scale, which included 4 statements (eg, “If it were up to you, to what extent would you want to use CareVirtue?”) and 5-point response scale from 1 (not at all) to 5 (a great deal) [30,31]. Third, we used the perceived usefulness survey, which includes 4 statements (eg, “Using CareVirtue would make it easier to perform my caregiving role”) and a 5-point response scale (1=strongly disagree to 5=strongly agree) [32]. Quantitative acceptability measures were collected from all primary caregiver participants. Secondary caregivers could opt in to complete these assessments by contacting the study team.

##### Qualitative Interviews

To provide context to the quantitative measures of acceptability, we conducted semistructured interviews with the primary caregivers, which focused on caregivers’ experiences with CareVirtue during the study period. The interview guide was developed with input from the full research team (Multimedia Appendix 3).

### Analysis Plan

#### Overview

We used Python 3.8 (Python Software Foundation) to compute descriptive statistics and conduct statistical analyses for all quantitative data. Qualitative data were coded using Microsoft Excel. Analyses related to research questions are described in detail below.

#### Who Used CareVirtue?

To determine participant characteristics, we computed descriptive statistics from demographic survey responses.

#### How Did Caregivers Use CareVirtue?

To assess usage heterogeneity, we computed descriptive statistics for each of the 8 usage characteristics. We then used k-means clustering to cluster care networks into “user types,” where each care network is represented by an 8-dimensional usage vector. The number of clusters, k, was varied from 1 to 20 and the elbow method was used to select the final k value: 8. See Multimedia Appendix 4 for more details.

#### How Did Caregivers Perceive CareVirtue Acceptability?

To assess perceptions of usability and usefulness, we computed descriptive statistics for the SUS, Behavioral Intention Scale, and Perceived Usefulness Scale. We also computed Pearson correlation coefficient for the weekly NASA-TLX to assess the change in overall caregiver workload over the study period and for the confidence survey to assess if confidence changed over the study period.

To further explore perceptions of usability and usefulness, we conducted a general content analysis of the interview transcripts.^53^ Three members of the research team (PL, SN, and AL) with training in human factors engineering reviewed all transcripts and identified initial categories related to CareVirtue usability and usefulness, with 2 team members coding each transcript. Coders met weekly to discuss codes and resolve discrepancies, which were also discussed in a biweekly meeting with a senior research team member (NEW) with expertise in qualitative research and human factors engineering. The codebook was refined iteratively across the team-based discussions and the final codebook was applied across all transcripts using a team-based consensus process [33].

#### What Factors Were Associated With CareVirtue Use?

To explore the factors associated with CareVirtue use, we conducted a series of univariate analyses to assess the correlation between each of the 8 usage characteristics and each of the 14 variables from the demographic survey: the NASA-TLX score from each week, the confidence score from each week, the SUS score, the average behavioral intention score, and the average perceived usefulness score. For continuous variables, we computed Pearson correlation coefficient and the corresponding 95% CI to assess if the correlation was statistically significant (*P*=.05). For discrete variables, we first converted them into binary variables (if not already) by merging classes to ensure suitable sample sizes. Then, we used an unpaired *t* test to assess whether the difference between the average from each class was statistically significant. We were unable to perform multivariate analyses due to our limited sample size.

### Ethics Approval

Research ethics approval was granted by the Institutional Review Board at the University of Wisconsin-Madison (Protocol #2020-1035).

## Results

### Who Used CareVirtue?

We enrolled 51 primary caregivers of people living with ADRD (Table 1) and 61 secondary caregivers to use CareVirtue during the study period. Care networks ranged from 1 to 8 members. Primary caregivers were predominantly female (38/51, 75%), White (44/51, 86%), married (37/51, 73%), college educated (36/51, 71%), and were, on average, 60.3 (SD 9.8) years of age. Care recipients were also primary female (34/51, 67%) and White (45/51, 88%), with an average age of 79.2 (SD 10.6). Care networks were located in both Wisconsin (29/51, 57%) and California (19/51, 37%), with 18% (9/51) living in a rural area. During the study period, 4 primary caregivers dropped out because of care recipient death (n=2) and personal situations (n=2). We were unable to reach 6 primary caregiver participants for the posttrial visit. A total of 12 secondary caregiver participants completed the postuse survey.

**Table 1:** Summary of primary caregiver and care recipient characteristics.

Characteristic			Primary caregivers (n=51)		Care recipients (n=51)
Female gender, n (%)			38 (75)		34 (67)
Age (years), mean (SD)			60.3 (9.8)		79.2 (10.6)
**Race/ethnicity, n (%)**					
	Asian	2 (4)		2 (4)	
	Black or African American	1 (2)		1 (2)	
	Hispanic or Latinx	2 (4)		2 (4)	
	Native American or American Indian	1 (2)		0 (0)	
	Not reported	1 (2)		1 (2)	
	White	44 (86)		45 (88)	
**Marital status, n (%)**					N/A^a^
	Married or domestic partnership	37 (73)			
	Divorced	11 (22)			
	Single, never married	2 (4)			
	Widowed	1 (2)			
**Education, n (%)**					N/A
	Postcollege	19 (37)			
	4-year college	17 (33)			
	Technical school, vocational training, community college	10 (20)			
	High school diploma or equivalent	5 (10)			
**Employment, n (%)**					N/A
	Full-time	21 (41)			
	Retired	19 (37)			
	Part-time	7 (14)			
	Not working	4 (8)			
**Income, n (%)**					N/A
	≤US $19,000	1 (2)			
	US $20,000-39,000	2 (4)			
	US $40,000-59,000	8 (16)			
	US $60,000-79,000	4 (8)			
	US $80,000-99,000	6 (12)			
	≥US $100,000	18 (35)			
	Do not wish to answer	8 (16)			
**Location, n (%)**					N/A
	Wisconsin	29 (57)			
	California	19 (37)			
	Illinois	2 (4)			
	Virginia	1 (2)			
**Location type, n (%)**					N/A
	Urban	42 (82)			
	Rural	9 (18)			
**Relationship to caregiver, n (%)**			N/A		
	Parent			28 (55)	
	Spouse/Partner			20 (39)	
	Other relative			3 (6)	
**Distance to caregiver, n (%)**			N/A		
	In household			34 (67)	
	<20 minutes			12 (24)	
	20-60 minutes			2 (4)	
	>2 hours			3 (6)	
**Living situation, n (%)**			N/A		
	In a house			40 (78)	
	In a nursing home, retirement community, or other assisted living facility			9 (18)	

^a^N/A: not applicable.

### How Did Caregivers Use CareVirtue?

**Figure 5:**
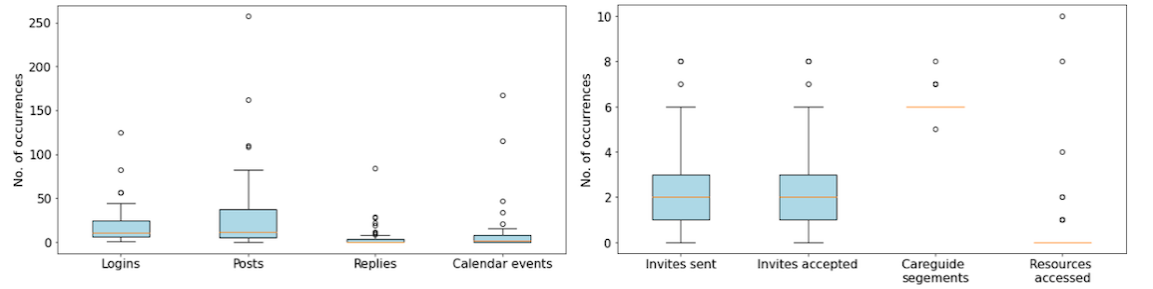
Box plots for each of the eight usage characteristics. They are separated into two plots due to differences in scale.

Figure 5 displays boxplots (across care networks) for each of the 8 usage characteristics. The average (SD) was 18.3 (22.4) log-ins, 32.5 (46.5) journal posts, 5.3 (13.2) journal post replies, 10.6 (28.5) calendar events, 2.2 (2.1) secondary caregiver invites sent, 2.2 (2.1) secondary caregiver invites accepted, 6.1 (0.4) care guide sections created, and 0.6 (1.9) resources accessed. The log-in data are not fully representative of actual use because users could remain logged in to CareVirtue depending on their preference to log out.

Table 2 presents the centroid (the mean values across all care networks in that cluster), cluster size, and a cluster label for each of the 8 clusters. The 8 clusters could be further reduced into 3 primary groups according to the degree of engagement with the platform. There was heterogeneity in how the most engaged care networks interacted with the platform; for example, 2 care networks made heavy use of the calendar feature (average of 141 events) with few posts (average of 41), while another care network made heavy use of the journal (257 posts) with only 1 calendar event.

**Table 2:** Cluster centroids for the 8 usage clusters identified by k-means. Each centroid component (eg, invites sent) represents the average across all care networks within that cluster.

Cluster description	Cluster size	Cluster centroid							
		Log-ins	Posts	Replies	Invites sent	Invites accepted	Calendar events	Care guide sections	Resources accessed
**Low engagement group**									
	23	10	13	1	1	1	3	6	0
**Moderate engagement group**									
Small care networks	12	13	25	2	3	2	6	6	0
Large care networks	7	19	40	11	6	6	3	6	0
**High engagement group**									
Log-in heavy	3	88	56	9	3	3	18	6	0
Balanced usage	2	30	53	14	4	4	18	6	9
Calendar focused	2	33	41	0	2	2	141	6	0
Posts and replies	1	15	162	84	3	3	16	8	2
Posts only	1	12	257	1	2	2	1	6	0

### How Did Caregivers Perceive CareVirtue Acceptability?

**Figure 6:**
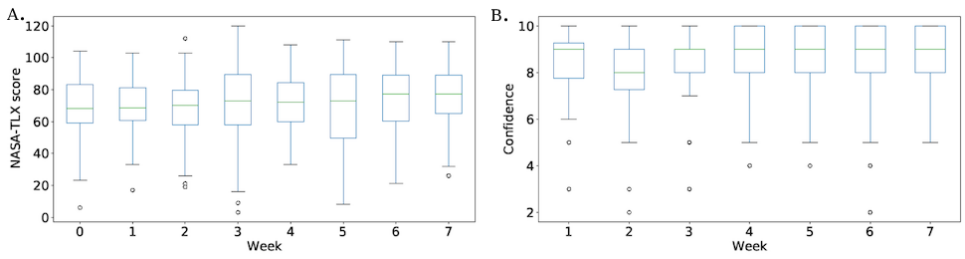
(A) Box plot for the weekly NASA TLX score. (B) Box plot for the weekly confidence (in using CareVirtue) survey.

We used the NASA TLX score to assess usability and usefulness in terms of caregivers’ perceptions of their caregiver workload. Figure 6A displays boxplots of the total NASA TLX score for each week from week 0 (before the study began) to 7 (the final week of the study). The average NASA TLX score increased over the duration of the study (*P*=.02; *r*=0.79, 95% CI 0.65-0.87). However, at an individual level the NASA TLX score only increased over the duration of the study for 3 primary caregivers, decreased for 2 caregivers, and did not change for the remaining 29 primary caregivers (17 were excluded due to missingness).

We used a confidence scale to assess CareVirtue usability. Figure 6B displays boxplots of the total confidence score for each week of the study. The average confidence score increased significantly over the duration of the study from a low of 7.8 in week 2 to a high of 8.9 in week 7 (*P*=.004; *r*=0.91, 95% CI 0.84-0.95). At an individual level, 7 primary caregivers saw a statistically significant increase (*P*<.05 in all cases; see Multimedia Appendix 5 for precise *P* values) in confidence, while the remaining 17 remained stable (27 were excluded due to missingness).

We also used the SUS to assess CareVirtue usability and the Behavioral Intention Scale and Perceived Usefulness Scale to assess usefulness. Multimedia Appendix 6 displays histograms for the Behavioral Intention Scale score, SUS score, and the perceived usefulness score for primary caregivers. The average (SD) was 3.3 (1.2) for the Behavioral Intention Scale and 72% (26/36) of primary caregivers reported an average score of at least 3, indicating an above average intention to use. The average (SD) was 81.8 (12.8) for the SUS score, indicating “good” usability and 3.4 (1.0) for perceived usefulness, suggesting above average perceived usefulness. For secondary caregivers the average (SD) was 2.9 (1.1) for the Behavioral Intention Scale (6/11, 55%, had an average score of at least 3), 77.9 (13.7) for the SUS, and 3.4 (0.8) for perceived usefulness (Multimedia Appendix 7).

Through analysis of the qualitative interviews, we identified 10 categories related to usefulness and 4 categories related to opportunities to improve usefulness (Table 3). Participants described CareVirtue as facilitating connection, exploration, and awareness. In addition, CareVirtue allowed for documentation and tracking of daily experiences and enabled emotional catharsis by facilitating the capture and review of significant moments in their relationship with the person living with ADRD. Additional facilitators of usefulness described by participants included centralization of information, coordination across the care network, and the privacy afforded by controlling user permissions. Participants further explained that using CareVirtue made them feel supported and reduced feelings of being overwhelmed. We also identified opportunities for improvement, which included increasing engagement such as creating a CareVirtue user support group; adding customization such as additional emoji options for journal posts; refining navigation such as expanding search types; and additional functionality such as document upload and storage.

**Table 3:** Categories of usefulness and opportunities for improvement with illustrative quotations.

Categories and subcategories			Description	Illustrative quotation	
**Usefulness**					
	Connection		Interaction across the care network	*The biggest thing was it [CareVirtue] allowed me to bring my brother and sister, both of whom live a thousand or more miles away, closer into the loop...at crucial times...there, you know, were some things going on, and they were actually getting the email reminders, and they were actually logging in and looking at my journal comments and responding in the journal.* [P45]	
	Documentation and Tracking		View, explore, and understand trends over time	*It helped me in terms of measuring my spouse’s progression over those two whole months. Because, you know, when you’re with someone every day, 24/7 almost, you may not notice the differences other people do. So, you know, I found that useful to go back and look at what I wrote, you know, a month ago. Because it appears the stage of the disease is, and*...*that it progresses could go, slow down and could speed up. So, it seemed like the progression was increasing and just help me quantify it to some degree in terms of what her capabilities were.* [P50]	
	Emotional Catharsis		Capture, share, and recollect experiences and important moments in the relationship with the person living with ADRD	*But the other day he said, you know, I love you. You’re my favorite. And he hadn’t said I love you to me in like, I don’t know, a few years*...*but to document a moment like that as a way for me to kind of cathartically capture those moments and have those to look back on.* [P13]	
	Awareness		Real-time understanding of daily care experiences and status of the caregiver and person living with ADRD	*[CareVirtue] also allowed me to let my kids know what was going on the same time it was happening, as far as what, you know, when I was doing the journaling. It also helped my kids to be able to see probably a really good picture of all the different aspects of what their mom is going through.* [P15]	
	Centralization and Organization		Communication, coordination, documentation, and tracking in one location	*So, with the CareVirtue, having one spot, like if I’m going to communicate, I’m going to put it in there, and then everybody can just go to that spot to look for the information...people can log-in and just have their notifications and know that stuff was going on. And it would be one step there versus me trying to figure out how to get, you know, am I in the right [text message] thread, which information needs to go to who?* [P26]	
	Coordination		Seamless, high-quality transitions of care	*But the app [CareVirtue] was really helpful, because before a person came in for their quote, unquote, shift time, they can have advance information about how things were before they came, so they could be kind of prepared.* [P32]	
	Introspection		Self-exploration of feelings, care strategies, and goals	*[CareVirtue] just helped me to identify, I guess, where, how I was feeling and what my plan was for going forward.* [P16]	
	Privacy		Customizable permissions on a secure platform focused only on care	*[Before CareVirtue] we kind of send stuff through Messenger, which is not necessarily a secure, you know, thing, and this one [CareVirtue] is. So, yeah, it has just created something that was specifically and exclusively for her care, and that, you know, that was good.* [P1]	
	Reduce Burden		Reduce demands associated with communication, coordination, and documentation	*[CareVirtue] was really useful in terms of keeping a journal and telling everybody what was going on without having to call every single family member, so they could read [in CareVirtue] what was going on.* [P7]	
	Support		Accessible and responsive customer support	*When I had a question, I just hit the little blue bubble and it sent a note. And in absolutely no time somebody [from CareVirtue support]...answered the question or told me how to do what I was, needed to do...It just made it really helpful, really easy to reach out.* [P23]	
**Opportunities for improving usefulness**					
		Engagement	Provide additional interactive content such as private journaling space, a support group across CareVirtue users, and a daily checklist of care activities	*It’s very personal. Even though they are family members, it’s kind of like do I really want them to read about my inner thoughts about this, you know, because it could frighten them.* [P37]	
		Customization	Include additional customization options such as for reminders, and including more emoji options	*The [emoji] smiley or the sad or whatever, it makes you really think about...I would like it that you could put a couple options in there though [instead of only one]. Because, you know, I might start the entry out in one way and then key in another because it turned.* [P44]	
		Navigation	Expand search feature	*A filter to search, you know, for key words, or maybe not even key words, any word search, you know, any text search, I find immensely valuable.* [P12]	
		Functionality	Add functionality to include allowing for an account with multiple care receivers and document upload and storage	*It still lacks the ability for it to be the all-inclusive kind of filing cabinet that I personally need it to be.* [P26]	

### What Factors Were Associated With CareVirtue Use?

The following sociodemographic characteristics were associated with the number of posts: retired (average of 59.5 posts for retired caregivers as compared with 16.9 for nonretired caregivers), income (13 posts for those reporting >US $100K and 55.4 for those reporting <US $100K), relationship to care recipient (18.7 posts for child and 56.4 for partners/spouses), and living situation (44.7 for those who live with the care recipient and 13.1 for those who do not). Older care recipients were associated with fewer posts (*r*=–0.33, 95% CI –0.55 to –0.06).

The following workload characteristics were associated with the number of posts: NASA TLX score representing the perceived workload associated with the caregiving role from weeks 2 to 7 (*r*=0.37-0.46) and the total hours caregiving (*r*=0.38, 95% CI 0.11-0.60). In other words, higher perceived mental workload associated with the caregiving role and a greater number of hours spent caregiving were associated with more journal posts.

Regarding usability, we found that a higher SUS score (*r*=0.40, 95% CI 0.10-0.64) and a higher behavioral intention score (*r*=0.378, 95% CI 0.11-0.59) were associated with an increased number of posts.

We found that 3 demographic characteristics were associated with the number of log-ins. Retired caregivers had an average of 27.4 log-ins as compared with 11.3 for nonretired caregivers. Both primary caregiver age (*r*=0.31, 95% CI 0.03-0.54) and the total behavioral intention score (*r*=0.38, 95% CI 0.11-0.59) were associated with more log-ins. We found that a higher SUS score was associated with an increased number of secondary caregiver invites sent (*r*=0.33, 95% CI 0.05-0.57).

## Discussion

### Principal Findings

This study establishes the acceptability and feasibility of CareVirtue use among care networks of individuals living with dementia. The results indicate that CareVirtue was perceived as highly usable and useful, with caregivers indicating a range of ways CareVirtue was useful to them, including practically, organizationally, and emotionally. We found that retirees, spouses/partners of the care recipient, and those who live with the care recipient were more likely to post more frequently in the journal. CareVirtue use was not correlated with caregiver age or education level.

Participants were confident in using CareVirtue, with confidence increasing over time, which aligns with their reported perceptions of CareVirtue as highly useful and usable. The qualitative analysis revealed that CareVirtue is useful across multiple dimensions including reducing burden associated with logistics and organization; providing emotional and social support; and facilitating documentation, tracking, and awareness across the care network.

Interestingly, participants’ perceived workload associated with their caregiving role increased over the study period. It is possible that this result was reflective of the burden of participating in the study. However, we also found that higher caregiving workload was associated with more frequent use of the CareVirtue journal. It is also possible that this consistent increase in perceived caregiving workload was related to the increased burden and isolation associated with the COVID-19 pandemic during which our study took place [34,35]. A recent survey study [33] found that caregiver burden was not associated with caregivers’ reported intention to adopt a mobile health (mHealth) intervention. Our finding based on the engagement with the technology intervention, that increased caregiver workload was associated with increased journal posts, provides additional insight into factors that may be influencing caregivers’ adoption and use of technology interventions. This finding also points to specific components, such as the ability to journal about daily experiences and emotions, that may be more useful during times of higher workload.

We found that usage varied along 2 axes: total usage and type of usage. There was heterogeneity in how the most engaged care networks interacted with the platform; for example, 2 care networks made heavy use of the calendar event feature (average of 141 events) with few posts (average of 41), while another care network made heavy use of the journal (257 posts) with only 1 calendar event. These results confirm the importance of technology interventions that can account for caregiver heterogeneity [36,37]—that caregivers are diverse individuals who have wide-ranging experiences, needs, and contexts. The need for interventions responsive to caregiver heterogeneity has been further supported by the identified importance of multiple component interventions that are tailorable to specific caregiver needs [5,9-11]. Our findings expand on this by providing insights into the acceptability and feasibility of a technology intervention at the care network level. Like studies focused on primary caregiver technology interventions, our results highlight the importance of designing flexible, multicomponent interventions that allow care networks to tailor their engagement according to their needs and what is most helpful to them. Importantly, this could also allow for tailoring over time, in which care networks can shift engagement as their needs change across disease progression.

This study used a sample size that is much larger than is typical for a feasibility study [38]. Doing so gave us the opportunity to leverage data analytics to provide insight into how care networks engaged with the platform and provided depth to our understanding of what components caregivers find useful [28]. Survey studies have been useful in capturing self-reported perceptions related to adoption and intention to use [39,40]. Findings such as ours along with others such as Øksnebjerg et al [41] can complement and expand upon findings from self-report studies by exploring caregivers’ engagement behaviors. Given caregiver and care network heterogeneity along with the evidence demonstrating the importance of flexible and tailorable multicomponent interventions [3,12-19], there is a need for future research to continue to explore caregiver and care network engagement behavior with technology interventions to provide additional insight and begin to build an evidence base regarding how to optimally tailor interventions and support engagement according to individual caregiver and care network needs. To do this, future feasibility trials could strive to engage larger sample sizes, enabling the use of artificial intelligence and machine learning to increase personalization. Further, efficacy/effectiveness trials that typically enroll larger samples could be used to explore engagement. Doing so may allow for an increased understanding of the relationship between engagement with the technology intervention and the health outcomes.

Although the purpose of this study was not to determine the effectiveness of CareVirtue on caregiver outcomes, previous research has demonstrated the potential of technology interventions to improve caregiver outcomes [3,12-19]. Research suggests that the significant unmet needs associated with support for communication and coordination among the care network may contribute to the often-suboptimal outcomes experienced by caregivers such as increased stress and burden [20,24,37]. Further, our findings related to caregiving workload and journal use combined with our qualitative findings provide some initial indication that caregivers may experience positive effects such as reduced burden, increased social support, and increased quality of life from using CareVirtue. Our immediate plan for future research is to conduct a randomized clinical trial to test the hypothesis that CareVirtue reduces caregiver stress and burden and increases caregiver quality of life.

### Limitations

Our results should be considered in light of certain limitations. First, although our sample size was much larger than is typical for a feasibility study [38], the sample size should be considered when interpreting the univariate results, as it is possible that a small group of people could be driving our findings. Second, although we achieved enrollment of diversity in terms of income and location within the United States, participants were primarily White, married, college-educated women of 60 years of age who lived with the care recipient. Future work will endeavor to achieve a more sociodemographically diverse sample in terms of race/ethnicity, education, age, and distance from the person living with dementia. Third, although this study found a broad range of care network sizes consistent with previous literature [20], it is likely that the context of the COVID-19 pandemic may have reduced the number of in-home supports, which may have influenced the number of care network members using CareVirtue.

The results of this study establish the acceptability and feasibility of CareVirtue use among care networks of people living with ADRD. This study also highlights the importance of designing flexible, multicomponent interventions that allow care networks to tailor their engagement according to their needs and what is most useful to them. The results of this feasibility study will be used to improve CareVirtue feasibility and acceptability in preparation for a subsequent randomized trial to test CareVirtue’s effectiveness in improving caregiver outcomes.

## Appendix

Multimedia Appendix 1 Walkthrough of CareVirtue features.

Multimedia Appendix 2 CareVirtue Care Guide template.

Multimedia Appendix 3 Semi-structured interview guide.

Multimedia Appendix 4 Elbow plot for k-means clustering. Each care team is represented by an eight-dimensional vector with the following components: the number of logins, the number of journal posts, the number of journal post replies, the number of calendar events, the number of secondary caregiver invites sent, the number of secondary caregiver invites accepted, the number of Care Guide sections created, and the number of resources accessed through CareVirtue. We clustered all observations using k-means clustering, with a Euclidian distance metric and 10 random initializations. We varied k from 1 to 20 and used the elbow method (on the intra cluster distance) to choose the final k value. Figure A1 displays the intra cluster distance for each k value.

Multimedia Appendix 5 *P* values for caregiver-reported confidence using CareVirtue over the duration of the study for the 24 participants included in the analysis (27 participants were excluded due to missingness).

Multimedia Appendix 6 Histograms for primary caregiver responses to the behavioral intention scale, system usability scale, and perceived usefulness.

Multimedia Appendix 7 Histograms for secondary caregiver responses to the behavioral intention scale, system usability scale, and perceived usefulness scale.

## Abbreviations

ADRD: Alzheimer disease and related dementias
MARS: Mobile Application Rating Scale
mHealth: mobile health
NASA-TLX: National Aeronautics and Space Administration-Task Load Index
SUS: System Usability Scale

## References

[1] Alzheimer's Disease and Related Dementias. Centers for Disease Control and Prevention.

[2] Monica MM, Díaz-Santos M, Vossel K (2021). Alzheimer’s Association 2021: Facts and Figures Report. Mary S. Easton Center for Alzheimer’s Disease Research at UCLA.

[3] Boots LMM, de Vugt ME, van Knippenberg RJM, Kempen GIJM, Verhey FRJ (2014). A systematic review of Internet-based supportive interventions for caregivers of patients with dementia. Int J Geriatr Psychiatry.

[4] Boots LMM, Wolfs CAG, Verhey FRJ, Kempen GIJM, de Vugt ME (2015). Qualitative study on needs and wishes of early-stage dementia caregivers: the paradox between needing and accepting help. Int. Psychogeriatr.

[5] Werner NE, Stanislawski B, Marx K, Watkins D, Kobayashi M, Kales H, Gitlin L (2017). Getting what they need when they need it. Appl Clin Inform.

[6] (2012). Next Steps for Research on Informal Caregiving. National Institute on Aging.

[7] (2015). Recommendations from the NIH AD Research Summit 2015. National Institute on Aging.

[8] Borson S, Boustani MA, Buckwalter KC, Burgio LD, Chodosh J, Fortinsky RH, Gifford DR, Gwyther LP, Koren MJ, Lynn J, Phillips C, Roherty M, Ronch J, Stahl C, Rodgers L, Kim H, Baumgart M, Geiger A, Alzheimer's Association National Plan CareSupport Milestone Workgroup (2016). Report on milestones for care and support under the U.S. National Plan to Address Alzheimer's Disease. Alzheimers Dement.

[9] Hopwood J, Walker N, McDonagh L, Rait G, Walters K, Iliffe S, Ross J, Davies N (2018). Internet-Based Interventions Aimed at Supporting Family Caregivers of People With Dementia: Systematic Review. J Med Internet Res.

[10] Etxeberria I, Salaberria K, Gorostiaga A (2021). Online support for family caregivers of people with dementia: a systematic review and meta-analysis of RCTs and quasi-experimental studies. Aging Ment Health.

[11] Deeken F, Rezo A, Hinz M, Discher R, Rapp MA (2019). Evaluation of Technology-Based Interventions for Informal Caregivers of Patients With Dementia-A Meta-Analysis of Randomized Controlled Trials. Am J Geriatr Psychiatry.

[12] Chiu T, Marziali E, Colantonio A, Carswell A, Gruneir M, Tang M, Eysenbach G (2009). Internet-based caregiver support for Chinese Canadians taking care of a family member with alzheimer disease and related dementia. Can J Aging.

[13] Glueckauf RL, Ketterson TU, Loomis JS, Dages P (2004). Online support and education for dementia caregivers: overview, utilization, and initial program evaluation. Telemed J E Health.

[14] Bateman DR, Srinivas B, Emmett TW, Schleyer TK, Holden RJ, Hendrie HC, Callahan CM (2017). Categorizing Health Outcomes and Efficacy of mHealth Apps for Persons With Cognitive Impairment: A Systematic Review. J Med Internet Res.

[15] Godwin KM, Mills WL, Anderson JA, Kunik ME (2013). Technology-driven interventions for caregivers of persons with dementia: a systematic review. Am J Alzheimers Dis Other Demen.

[16] Martínez-Alcalá Claudia I, Pliego-Pastrana P, Rosales-Lagarde A, Lopez-Noguerola J, Molina-Trinidad EM (2016). Information and Communication Technologies in the Care of the Elderly: Systematic Review of Applications Aimed at Patients With Dementia and Caregivers. JMIR Rehabil Assist Technol.

[17] Marziali E, Garcia LJ (2011). Dementia caregivers' responses to 2 Internet-based intervention programs. Am J Alzheimers Dis Other Demen.

[18] Lewis ML, Hobday JV, Hepburn KW (2010). Internet-based program for dementia caregivers. Am J Alzheimers Dis Other Demen.

[19] van der Roest Henriëtte G, Meiland FJM, Jonker C, Dröes Rose-Marie (2010). User evaluation of the DEMentia-specific Digital Interactive Social Chart (DEM-DISC). A pilot study among informal carers on its impact, user friendliness and, usefulness. Aging Ment Health.

[20] Ponnala S, Werner NE (2021). Exploring Informal Caregiver Workload using a Macroergonomics Lens on Multiple Resources. Proceedings of the Human Factors and Ergonomics Society Annual Meeting.

[21] Friedman E, Kennedy D (2021). Typologies of Dementia Caregiver Support Networks: A Pilot Study. Gerontologist.

[22] Esandi Nuria, Nolan Mike, Alfaro Cristina, Canga-Armayor Ana (2018). Keeping Things in Balance: Family Experiences of Living With Alzheimer's Disease. Gerontologist.

[23] Neubert L, Gottschalk S, König Hans-Helmut, Brettschneider C (2022). Dementia care-giving from a family network perspective in Germany: A typology. Health Soc Care Community.

[24] Tang C, Chen Y, Cheng K, Ngo V, Mattison JE (2017). Awareness and handoffs in home care: coordination among informal caregivers. Behaviour & Information Technology.

[25] Werner NE, Brown JC, Loganathar P, Holden RJ (2022). Quality of Mobile Apps for Care Partners of People With Alzheimer Disease and Related Dementias: Mobile App Rating Scale Evaluation. JMIR Mhealth Uhealth.

[26] Wozney L, Freitas de Souza LM, Kervin E, Queluz F, McGrath PJ, Keefe J (2018). Commercially Available Mobile Apps for Caregivers of People With Alzheimer Disease or Other Related Dementias: Systematic Search. JMIR Aging.

[27] Block L, Gilmore-Bykovskyi A, Jolliff A, Mullen S, Werner NE (2020). Exploring dementia family caregivers' everyday use and appraisal of technological supports. Geriatr Nurs.

[28] Boutilier JJ, Craig T, Sharpe MB, Chan TCY (2016). Sample size requirements for knowledge-based treatment planning. Med Phys.

[29] Bangor A, Kortum PT, Miller JT (2008). An Empirical Evaluation of the System Usability Scale. International Journal of Human-Computer Interaction.

[30] Holden RJ, Karsh B (2009). A theoretical model of health information technology usage behaviour with implications for patient safety. Behaviour & Information Technology.

[31] Asan O, Holden RJ, Flynn KE, Murkowski K, Scanlon MC (2018). Providers' assessment of a novel interactive health information technology in a pediatric intensive care unit. JAMIA Open.

[32] Davis FD (1989). Perceived Usefulness, Perceived Ease of Use, and User Acceptance of Information Technology. MIS Quarterly.

[33] Barry CA, Britten N, Barber N, Bradley C, Stevenson F (1999). Using reflexivity to optimize teamwork in qualitative research. Qual Health Res.

[34] Aledeh M, Habib Adam P (2020). Caring for Dementia Caregivers in Times of the COVID-19 Crisis: A Systematic Review. AJNR.

[35] Bacsu J, O'Connell ME, Cammer A, Azizi M, Grewal K, Poole L, Green S, Sivananthan S, Spiteri RJ (2021). Using Twitter to Understand the COVID-19 Experiences of People With Dementia: Infodemiology Study. J Med Internet Res.

[36] Young HM, Bell JF, Whitney RL, Ridberg RA, Reed SC, Vitaliano PP (2020). Social Determinants of Health: Underreported Heterogeneity in Systematic Reviews of Caregiver Interventions. Gerontologist.

[37] Marcum C, Ashida S, Koehly L (2020). Primary Caregivers in a Network Context. J Gerontol B Psychol Sci Soc Sci.

[38] Julious SA (2005). Sample size of 12 per group rule of thumb for a pilot study. Pharmaceut. Statist.

[39] Wang J, Fu Y, Lou V, Tan S, Chui E (2021). A systematic review of factors influencing attitudes towards and intention to use the long-distance caregiving technologies for older adults. Int J Med Inform.

[40] Mendez KJW, Budhathoki C, Labrique AB, Sadak T, Tanner EK, Han HR (2021). Factors Associated With Intention to Adopt mHealth Apps Among Dementia Caregivers With a Chronic Condition: Cross-sectional, Correlational Study. JMIR Mhealth Uhealth.

[41] Øksnebjerg Laila, Woods B, Ruth K, Lauridsen A, Kristiansen S, Holst HD, Waldemar G (2020). A Tablet App Supporting Self-Management for People With Dementia: Explorative Study of Adoption and Use Patterns. JMIR Mhealth Uhealth.

