# Correction: A Web-Based Platform (CareVirtue) to Support Caregivers of People Living With Alzheimer Disease and Related Dementias: Mixed Methods Feasibility Study

**DOI:** 10.2196/41912

**Published:** 2022-09-14

**Authors:** Justin J Boutilier, Priya Loganathar, Anna Linden, Eleanore Scheer, Sofia Noejovich, Christian Elliott, Matthew Zuraw, Nicole E Werner

**Affiliations:** 1 Department of Industrial and Systems Engineering University of Wisconsin-Madison Madison, WI United States; 2 Department of Engineering Systems and Environment University of Virginia Charlottesville, VA United States; 3 Whiplash Technology, Inc Palm Springs, CA United States; 4 Department of Health and Wellness Design Indiana University School of Public Health-Bloomington Bloomington, IN United States

In “Web-Based Platform (CareVirtue) to Support Caregivers of People Living With Alzheimer Disease and Related Dementias: Mixed Methods Feasibility Study” (JMIR Aging 2022;5(3):e36975), the authors noted an error in the attribution of affiliations. Authors Justin J Boutilier, Priya Loganathar, Anna Linden, and Sofia Noejovich had the following incorrect affiliation attributed to their names:

Department of Health and Wellness Design, Indiana University School of Public Health-Bloomington, Bloomington, IN, United States

The correct affiliation for these authors, which did not appear in the original article, is as follows:

Department of Industrial and Systems Engineering, University of Wisconsin-Madison, Madison, WI, US

This affiliation appears in the corrected article as affiliation 1 and the remaining affiliations have been reordered accordingly.

The full list of authors and affiliations in the originally published article was as follows:

Justin J Boutilier^1^, PhD; Priya Loganathar^1^, MS; Anna Linden^1^, MSc; Eleanore Scheer^2^, BSc; Sofia Noejovich^1^, BSc; Christian Elliott^3^, BSc; Matthew Zuraw ^3^, MBA; Nicole E Werner^1^, PhD

^1^Department of Health and Wellness Design, Indiana University School of Public Health-Bloomington, Bloomington, IN, United States

^2^Department of Engineering Systems and Environment, University of Virginia, Charlottesville, VA, United States

^3^Whiplash Technology, Inc, Palm Springs, CA, US

The full list of authors and affiliations in the corrected version of the article appears as follows:

Justin J Boutilier^1^, PhD; Priya Loganathar^1^, MS; Anna Linden^1^, MSc; Eleanore Scheer^2^, BSc; Sofia Noejovich^1^, BSc; Christian Elliott^3^, BSc; Matthew Zuraw^3^, MBA; Nicole E Werner^4^, PhD

^1^Department of Industrial and Systems Engineering, University of Wisconsin-Madison, Madison, WI, US

^2^Department of Engineering Systems and Environment, University of Virginia, Charlottesville, VA, US

^3^Whiplash Technology, Inc., Palm Springs, CA, US

^4^Department of Health and Wellness Design, Indiana University School of Public Health-Bloomington, Bloomington, IN, US

The correction will appear in the online version of the paper on the JMIR Publications website on September 14, 2022, together with the publication of this correction notice. Because this was made after submission to PubMed, PubMed Central, and other full-text repositories, the corrected article has also been resubmitted to those repositories.

