# Supplementary material for: A Web-Based Platform (CareVirtue) to Support Caregivers of People Living With Alzheimer Disease and Related Dementias: Mixed Methods Feasibility Study
Source: JMIR Aging. 2022 Aug 4;5(3):e36975. doi: 10.2196/36975 (PMC9389379; doi:10.2196/36975)
Supplement: Multimedia Appendix 2 [file aging_v5i3e36975_app2.pdf]

Created on: 04/26/2022

Care Guide for Samuel Brown

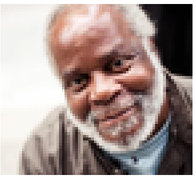

Preferred Name Sam

Important Things To Know

Allergic to shellfish, including shrimp.

Needs help walking down stairs.

Prefers to sleep in his recliner.

Likes jazz and bluegrass music.

Golden State Warriors and University of Kentucky basketball fan.

Activities of Daily Life (ADLs)

Instrumental Activities	Independent	Needs Help	Cannot Do
**Shopping (groceries, etc.)**			●
Needs to be accompanied to store with a written list. JPM note.			
**Cooking and meal prep**		●	
No open flame cooking. Microwave okay.			
**Managing medications**		●	
Needs daily reminders. Written schedule is on the fridge.			
**Transportation (driving, etc.)**		●	
Sam is happy to be a car passenger. He has a bus pass for short trips to the local parks.			
**Managing finances**			●
Daughter Anne handles finances.			
**Housekeeping (laundry, etc.)**	●		
Notes...			
**Use phone, computer, remotes**		●	
Needs reminding on using Chrome browser bookmarks.			
**Basic Activities**	**Independent**	**Needs Help**	**Cannot Do**

**Bathing**	●		
Notes...			
**Dressing**	●		
Notes...			
**Grooming**	●		
Notes...			
**Oral Care**	●		
Notes...			
**Eating**	●		
Notes...			
**Walking**	●		
Sam uses a cane as needed. Usually on bad weather days.			
**Climbing stairs**		●	
Sam has trouble walking *down* stairs. He should always go slow, and hold the railing.			
**Toileting**	?	?	?
Notes...			

Food and Drink

What does the care recipient like for his/her meals, and what foods/drinks should be avoided?

Breakfast

Coffee: black with one Splenda, Greek yogurt with granola, fresh fruit, cottage cheese mixed with cinnamon and peanuts. An occasional omelet with ham, onion, spinach, red and yellow bell peppers. Add a slice of avocado on top.

Lunch

Light lunches with some protein: chicken salad, tuna salad, chicken with steamed vegetables, chicken soup.

Dinner & Evening

Chili with cheese, onions and crackers, grits, meatloaf, soup, beans and corn bread, various Chinese dishes, cooked seafood

Evening: A shot of bourbon on the rocks or neat, an occasional beer

Food Allergies/Food Dislikes

Allergic to shellfish, including shrimp

Fried chicken, it's tasty but makes me nauseous every time.

Cranberry sauce, sushi, cooked spinach, hominy, over-cooked vegetables, tomato based pasta sauces.

Grooming & Comfort

Grooming Preferences

I like to keep my hair clean and my teeth brushed. It used to be important to me to be clean shaven but any more I really don't care except it does interfere with my bipap mask seal.

Keeping a warm blanket near would be great. I would like the occasional shot of bourbon.

Bathing & Clothing Preferences

Warm up the bathroom before a shower. I prefer a shower during daytime.

I like being comfortable, pajama bottoms and a T-shirt are awesome.

Sleeping Preferences

Unless my tendencies change, I prefer to spend most of my sleep time in my recliner rather than the bed.

Temperature Preferences

I like to keep the house at 72 degrees.

Triggers/Dislikes

I refuse to take a shower at night or when its cold (same reason really, the cold really affects me and at night I want to sleep, not get more awake). In general, I have come to hate the cold, it sends me into a deep mental fog that becomes difficult to recover from.

I really dislike having my face patted or rubbed or my ears stoked. Patting my face really pisses me off.

Music and TV

Music Preferences

Jimmy Buffett, Weird Al, Scottish banjo, jazz, oldies (50's, 60's). Likes 50s country music as well.

TV and Movie Preferences

Basketball, especially when UK is playing. I have always enjoyed comedies on tv as well as many dramas. Love any Mel Brooks movie.

I can't stand watching the news these days. I suppose I should stay informed, but it all irritates me to no end. I enjoy watching late night shows.

Triggers/Dislikes

Hard rock, most classical, gangster rap, any genre where the lyrics are screamed. TV with a lot of angst, whiny characters, shows that make the parents look like bumbling fools. Reality TV shows glamorizing horrible childhood behavior or adults who use everyone else to get through life.

Biography

About Samuel Brown

I was born and raised in a small east Kentucky town. I've always loved the water and exploring, so I joined the U.S. Navy in 1965. I retired as a Chief Petty Officer in 1987. After that, I was an engineering manager at Honeywell for 15 years.

I have two "kids" – Anne and Caleb – and three incredible grandchildren, Joshua, Caleb Jr, and Caroline, who I don't get to see as much as I'd like.

One of my greatest joys as a parent was being able to show my children the world. I believe that opening a child's eyes to the wonders of our planet, and the differences in language, customs, and history is important. Caleb was always interested in the history of countries we visited, especially in Europe. Anne soaked up different languages with ease, a skill that I know didn't come from me!

I love watching sports, especially basketball. I've adopted the Golden State Warriors, but I'm still a big University of Kentucky basketball fan. Go Wildcats!! New text.

Activities & Hobbies

Activity and Hobby Preferences

Woodworking, watching concerts, family gatherings

Surfing the web, online games.

Tending my garden, although I am needing more help with the latter lately. I like being outside on nice days, but not around large crowds.

Triggers/Dislikes

Most activities with large crowds and overwhelming noise.

In general, I dislike being in public for long periods, but I can handle going to the grocery or pharmacy. I have a hard time even riding around in the car for too long, need things quiet and normal, routine.
