# Supplementary material for: A Web-Based Platform (CareVirtue) to Support Caregivers of People Living With Alzheimer Disease and Related Dementias: Mixed Methods Feasibility Study
Source: JMIR Aging. 2022 Aug 4;5(3):e36975. doi: 10.2196/36975 (PMC9389379; doi:10.2196/36975)
Supplement: Multimedia Appendix 3 [file aging_v5i3e36975_app3.docx]

## Appendix 1. Semi-structured interview guide.

I would first like to ask you some questions about your experience using CareVirtue:

1. Thinking about your experience using CareVirtue over the last few months, how was it useful to you in your daily caregiving?

Prompts:

• What features did you like the most?

• What features made it less useful to you, that you liked the least?

2. What was your experience like with fitting CareVirtue into your day-to-day life?

Prompts:

• What parts of CareVirtue did you use on a daily basis? What made you return to use CareVirtue?

• What made you feel confident in using CareVirtue in your day-to-day life? What made you feel less confident?

• What changes did you make to your daily routines to use CareVirtue? [probe about nature of changes and whether they were positive or negative]

• What additional functionality would make CareVirtue more impactful for you and other caregivers?

• How did CareVirtue fit with the tools and resources you & your care team were already using?

• What parts did you want to use but did not use as often? Why?

3. Care team/secondary caregivers?

Prompts:

• What functions of CareVirtue were most useful in interacting with your care team?

• How could CareVirtue have been more useful to your care team interactions?

• How has using CareVirtue influenced the involvement of the other care team members?

• What kind of feedback have you received from your care team on the CareVirtue app?

4. How would you describe CareVirtue to friends, family, and other people in your life?
