# Supplementary figures and images for: A Web-Based Platform (CareVirtue) to Support Caregivers of People Living With Alzheimer Disease and Related Dementias: Mixed Methods Feasibility Study
Source: JMIR Aging. 2022 Aug 4;5(3):e36975. doi: 10.2196/36975 (PMC9389379; doi:10.2196/36975)

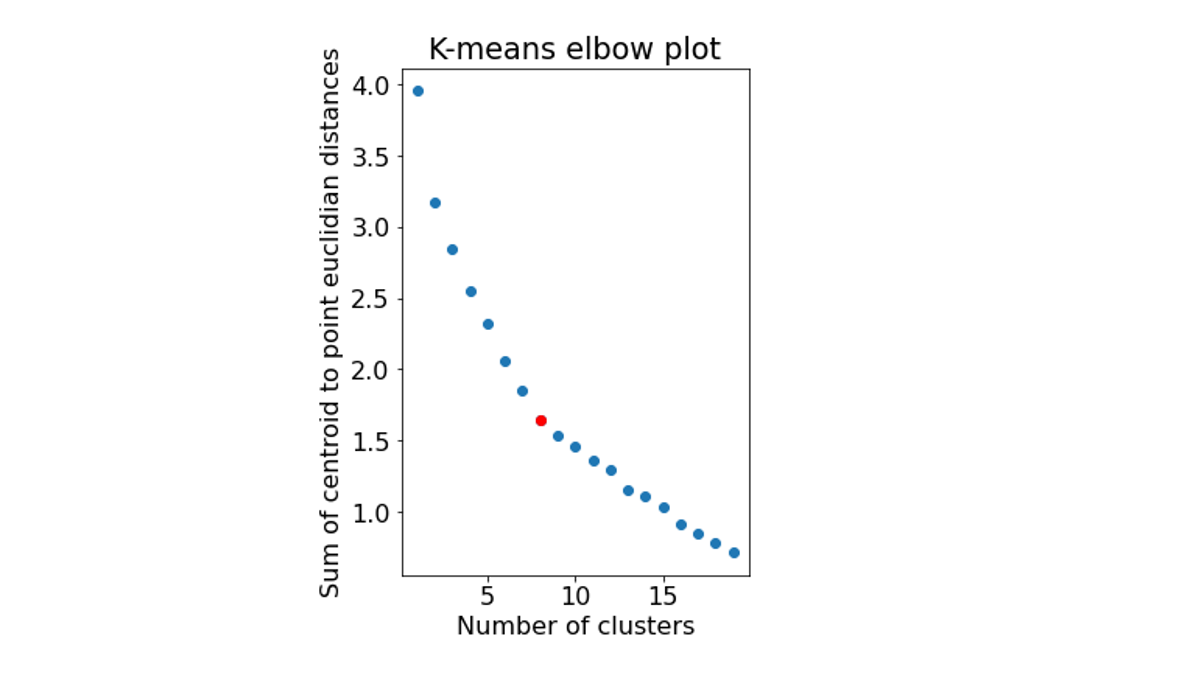

Supplement: Multimedia Appendix 4 [file aging_v5i3e36975_app4.png]

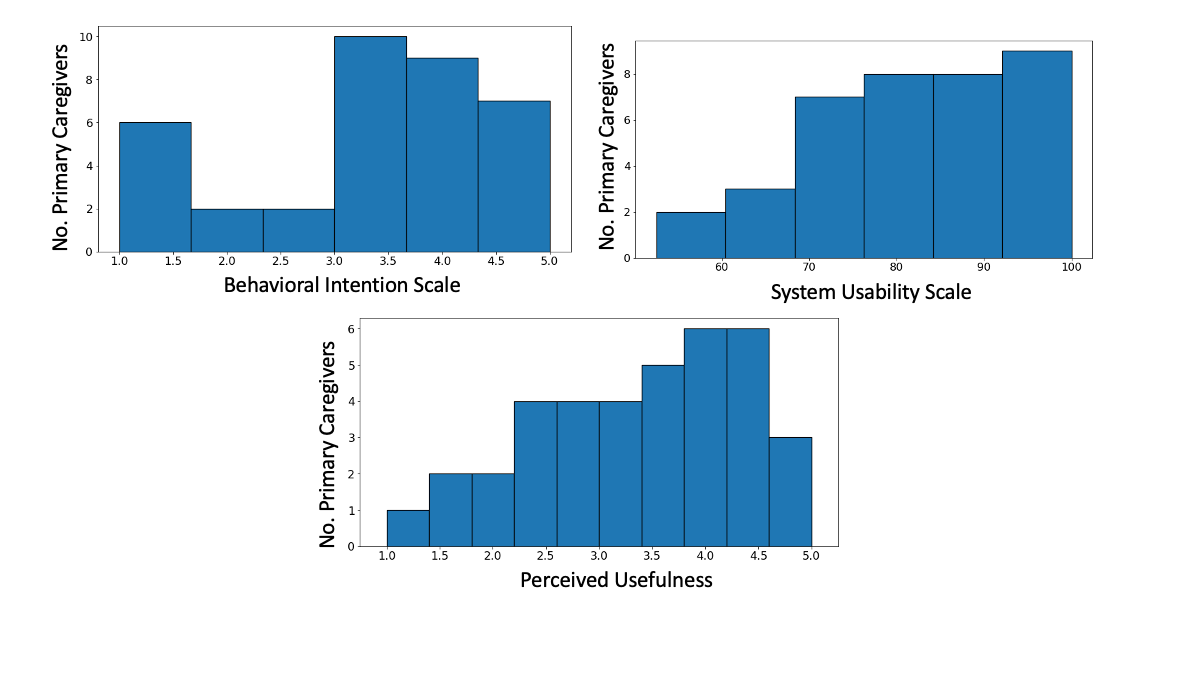

Supplement: Multimedia Appendix 6 [file aging_v5i3e36975_app6.png]

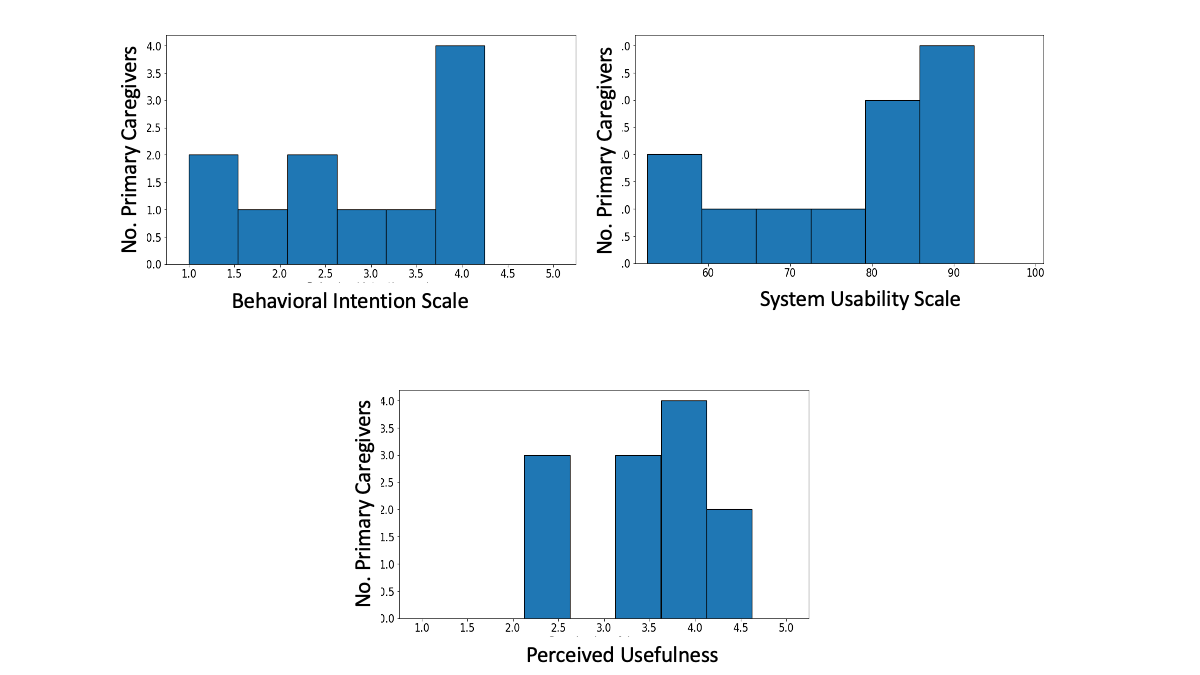

Supplement: Multimedia Appendix 7 [file aging_v5i3e36975_app7.png]
