# Supplementary material for: A Web-Based Platform (CareVirtue) to Support Caregivers of People Living With Alzheimer Disease and Related Dementias: Mixed Methods Feasibility Study
Source: JMIR Aging. 2022 Aug 4;5(3):e36975. doi: 10.2196/36975 (PMC9389379; doi:10.2196/36975)
Supplement: Multimedia Appendix 5 [file aging_v5i3e36975_app5.docx]

| **Pearson correlation coefficient** | **p-value** |
| --- | --- |
| 0.857 | 0.0136* |
| 0.408 | 0.3632 |
| 0.408 | 0.3632 |
| 0.894 | 0.0066* |
| 0.408 | 0.3632 |
| 0.408 | 0.3632 |
| 0.000 | 1.0000 |
| 0.567 | 0.1844 |
| -0.316 | 0.4896 |
| 0.217 | 0.6410 |
| 0.474 | 0.2822 |
| 0.866 | 0.0117* |
| 0.204 | 0.6606 |
| 0.000 | 1.0000 |
| 0.791 | 0.0343* |
| 0.000 | 1.0000 |
| 0.632 | 0.1275 |
| 0.612 | 0.1438 |
| 0.869 | 0.0112* |
| 0.392 | 0.3841 |
| 0.406 | 0.3667 |
| 0.567 | 0.1844 |
| 0.883 | 0.0085* |
| 0.791 | 0.0343* |

*Statistically significant increase at *P*<.05
